# Design and evaluation of magnetic-targeted bilosomal gel for rheumatoid arthritis: flurbiprofen delivery using superparamagnetic iron oxide nanoparticles

**DOI:** 10.3389/fphar.2024.1433734

**Published:** 2024-08-23

**Authors:** Rayan Y. Mushtaq, Nimbagal Raghavendra Naveen, Krishna Jayanth Rolla, Humood Al Shmrany, Sameer Alshehri, Ahmad Salawi, Mallesh Kurakula, Majed A. Alghamdi, Waleed Y. Rizg, Rana B. Bakhaidar, Walaa A. Abualsunun, Khaled M. Hosny, Abdulmohsin J. Alamoudi

**Affiliations:** ^1^ Department of Pharmaceutics, College of Clinical Pharmacy, Imam Abdulrahman Bin Faisal University, Dammam, Saudi Arabia; ^2^ Department of Pharmaceutics, Sri Adichunchanagiri College of Pharmacy, Adichunchanagiri University, Mandya, Karnataka, India; ^3^ Department of Data Analytics, Global Technical Software Service, Inc. (GTSS), Hamilton, NJ, United States; ^4^ Department of Medical Laboratory, College of Applied Medical Sciences, Prince Sattam Bin Abdulaziz University, Alkharj, Saudi Arabia; ^5^ Department of Pharmaceutics and Industrial Pharmacy, College of Pharmacy, Taif University, Taif, Saudi Arabia; ^6^ Department of Pharmaceutics, College of Pharmacy, Jazan University, Jazan, Saudi Arabia; ^7^ Thermo Fisher Scientific, Bend, OR, United States; ^8^ Department of Pharmaceutics, Faculty of Pharmacy, King Abdualziz University, Jeddah, Saudi Arabia; ^9^ Department of Pharmacology and Toxicology, Faculty of Pharmacy, King Abdulaziz University, Jeddah, Saudi Arabia

**Keywords:** quality by design, design of experiments, flubiprofen-loaded bilosomes, superparamagnetic iron oxide nanoparticles, targeted drug delivery, osteoarthritis management

## Abstract

**Introduction:**

The study aimed to systematically enhance the fabrication process of flurbiprofen-loaded bilosomes (FSB) using Quality by Design (QbD) principles and Design of Experiments (DOE). The objective was to develop an optimized formulation with improved entrapment efficiency and targeted drug delivery capabilities.

**Methods:**

The optimization process involved applying QbD principles and DOE to achieve the desired formulation characteristics. Superparamagnetic iron oxide nanoparticles (SPIONs) were incorporated to impart magnetic responsiveness. The size, entrapment efficiency, morphology, and *in vitro* release patterns of the FSB formulation were evaluated. Additionally, an *in situ* forming hydrogel incorporating FSB was developed, with its gelation time and drug release kinetics assessed. *In vivo* studies were conducted on osteoarthritic rats to evaluate the efficacy of the FSB-loaded hydrogel.

**Results:**

The optimized FSB formulation yielded particles with a size of 453.60 nm and an entrapment efficiency of 91.57%. The incorporation of SPIONs enhanced magnetic responsiveness. Morphological evaluations and *in vitro* release studies confirmed the structural integrity and sustained release characteristics of the FSB formulation. The *in situ* forming hydrogel exhibited a rapid gelation time of approximately 40 ± 1.8 s and controlled drug release kinetics. *In vivo* studies demonstrated a 27.83% reduction in joint inflammation and an 85% improvement in locomotor activity in osteoarthritic rats treated with FSB-loaded hydrogel.

**Discussion:**

This comprehensive investigation highlights the potential of FSB as a promising targeted drug delivery system for the effective management of osteoarthritis. The use of QbD and DOE in the formulation process, along with the integration of SPIONs, resulted in an optimized FSB formulation with enhanced entrapment efficiency and targeted delivery capabilities. The *in situ* forming hydrogel further supported the formulation’s applicability for injectable applications, providing rapid gelation and sustained drug release. The *in vivo* results corroborate the formulation’s efficacy, underscoring its potential for improving the treatment of osteoarthritis.

## 1 Introduction

Rheumatoid Arthritis (RA) poses a significant challenge in the domain of autoimmune disorders, impacting millions globally and placing substantial burdens on patients and healthcare systems alike. Characterized by persistent inflammation, synovial hyperplasia, and progressive joint degradation, RA not only diminishes patients’ quality of life but also carries the risk of long-term disability if left unaddressed (Radu and Bungau, 2021). Contemporary therapeutic approaches encompass a range of interventions such as nonsteroidal anti-inflammatory drugs (NSAIDs), disease-modifying antirheumatic drugs (DMARDs), and biologics. These strategies aim to mitigate symptoms, slow down the progression of the disease, and improve joint function. However, these treatments frequently elicit systemic side effects, demonstrate incomplete efficacy, and necessitate continuous and protracted administration (Nogueira et al., 2016).

The intricacies of RA pathogenesis necessitate innovative strategies to overcome the limitations of traditional therapies (Rabiei et al., 2021). Nanotechnology, renowned for its capacity to manipulate materials at the nanoscale, has unveiled novel avenues for precision medicine in rheumatoid arthritis (RA) management. Specifically, the integration of superparamagnetic iron oxide nanoparticles (SPIONs) into drug delivery systems presents a compelling opportunity to revolutionize RA treatment (Chen et al., 2017). SPIONs, nano-sized particles endowed with distinctive magnetic properties such as superparamagnetism, are composed of iron oxide and demonstrate biocompatibility. Beyond SPIONs, several other types of nanoparticles are emerging as promising candidates for RA therapy. Liposomes, for instance, are spherical vesicles with a phospholipid bilayer that can encapsulate both hydrophilic and hydrophobic drugs, enhancing their stability and bioavailability. Polymeric nanoparticles, made from biodegradable polymers like PLGA (poly (lactic-co-glycolic acid)), offer controlled and sustained drug release, reducing the frequency of dosing and improving patient compliance. Gold nanoparticles, known for their ease of surface modification and unique optical properties, can be utilized for targeted drug delivery and photothermal therapy. Additionally, dendrimers, which are highly branched, star-shaped macromolecules, provide multiple surface functionalities for drug attachment, allowing for precise targeting and controlled drug release. These diverse nanoparticle systems illustrate the broad potential of nanotechnology in advancing RA treatment by improving drug delivery, targeting inflamed tissues, and minimizing systemic side effects (Chen et al., 2017).

Guidable to specific sites using external magnetic fields, SPIONs find utility in targeted drug delivery, imaging (notably in MRI as contrast agents), hyperthermia therapy, and as versatile platforms for diagnostics and therapeutics in biomedical and nanomedicine research. Leveraging their unique magnetic characteristics, SPIONs can facilitate targeted drug delivery, potentially enhancing therapeutic efficacy while minimizing systemic side effects. Magnetic targeting enables the precise localization of drug-loaded nanoparticles to inflamed joints, augmenting drug concentration at the target site and mitigating exposure to healthy tissues—a particularly promising prospect for RA, where localized inflammation is a hallmark feature (Dulińska-Litewka et al., 2019).

In this context, our study pioneers an innovative approach for managing RA by amalgamating the anti-inflammatory attributes of flubiprofen with the magnetic targeting prowess of SPIONs within a bilosomes-loaded gel system. Bilosomes, lipid-based vesicles modified with bile salts, are ideally suited as carriers for drug delivery due to their heightened stability and drug encapsulation efficiency. Through the encapsulation of flubiprofen within bilosomes and the incorporation of SPIONs, our investigation endeavors to devise a novel therapeutic strategy that confronts the challenges associated with RA management. This envisioned system harbors the potential to refine drug delivery precision, mitigate systemic side effects, and bolster therapeutic outcomes for RA patients. Through our research endeavors, we aim to furnish valuable insights that propel the frontier of nanomedicine, advancing the effective and targeted management of Rheumatoid Arthritis.

## 2 Materials and methodology

### 2.1 Materials

Flurbiprofen (FBP) was generously provided by Sun Pharmaceutical Industries LTD. (Mumbai, India). Sodium taurocholate hydrate (STC) was procured from Sigma-Aldrich, United States. Sorbitan monostearate (Span^®^ 60) and Hyaluronic acid (HA) were sourced from Yarrow Chemicals, India. Synperonic™ PE/F 127 (PE/F127) and cholesterol (CHL) were acquired from Sigma-Aldrich Corp., St. Louis, MO, United States. Additionally, carrageenan, polyoxyethylene sorbitan monostearate (Tween^®^ 60), and cellulose dialysis membrane (with a molecular weight cutoff of 12,000–14,000) were purchased from the same supplier. All other chemicals used were of analytical grade.

### 2.2 Methods

The formulation process involved several critical steps, each meticulously designed to achieve the desired characteristics of the final product. Firstly, the preparation of FBP loaded bilosomes was undertaken, where FBP was effectively encapsulated within bilosomes through a well-established method ensuring efficient loading and stability. Following this, superparamagnetic iron oxide nanoparticles (SPIONs) were synthesized using a controlled chemical procedure to produce nanoparticles with optimal magnetic properties and size distribution. Subsequently, SPIONs were integrated into the FBP-loaded bilosomes, resulting in the formation of FBP/SPION loaded bilosomes (Dimitrov et al., 1984; Dai et al., 2013; Salama et al., 2022a). This integration required precise handling to maintain the integrity and functionality of both FBP and SPIONs (Abdelbary et al., 2016; El-Ridy et al., 2017). The characterization of the FBP/SPION loaded bilosomes (FSB) was then performed, measuring key parameters such as particle size, polydispersity index (PDI), and zeta potential to confirm the uniformity, stability, and surface charge of the formulations. Finally, the preparation of FSB *in situ* forming hydrogels was carried out, formulating the hydrogels to enable a responsive and sustained release of the encapsulated FBP and SPIONs, thus optimizing the therapeutic potential of the final product.

#### 2.2.1 Preparation of FBP-loaded bilosomes

The preparation of flurbiprofen (FBP)-loaded bilosomes was conducted using the thin film hydration method, with modifications applied to both the type and amount of bile salt, as well as the surfactant type. Initially, a precise quantity of FBP (16 mg), surfactant (Span 60), and 25 mg of cholesterol (CHL) were dissolved in 10 mL of chloroform, along with varying amounts of the chosen bile salt (sodium taurocholate hydrate–STC). This solution underwent sonication for 10 min to achieve a clear organic solution. Subsequently, the solution was transferred to a 250-mL round bottom flask and evaporated slowly at 60°C under reduced pressure using a rotary evaporator for 30 min at 120 rpm until a thin, completely dry film was obtained. The hydration of the dry film was performed with 10 mL of phosphate-buffered saline (PBS) at pH 7.4 using the same equipment under normal pressure. This hydration process involved spinning the flask in a water bath at 60°C for 30 min at 150 rpm, with the addition of glass beads to enhance nanovesicle yield. The resulting large vesicle dispersion underwent further size reduction through sonication for 3 min in a bath sonicator at 25°C. Finally, to ensure complete vesicle annealing and drug partitioning between the aqueous core and bilayer, the refined dispersion was allowed to equilibrate overnight at 4°C (Muzzalupo et al., 2011). This meticulous methodology ensures the successful fabrication of FBP-loaded bilosomes with optimized characteristics for drug delivery.

The optimization of flurbiprofen (FBP)-loaded bilosome preparation was carried out using a statistical methodology. Independent variables under consideration included the concentrations of surfactant (Span 60) (X1) and bile salt concentration (STC) (X2). Each factor’s low, medium, and high levels were represented by the codes −1.414, −1, 0, + 1, and +1.414. Dependent variables such as entrapment effectiveness (PS-Y1) and entrapment efficiency (EE-Y2) were utilized to evaluate the outcomes of the optimization process (please refer to Table 1 for further details).

**TABLE 1:** Central-composite optimization design for FBP-loaded bilosomes [Span 60 and STC conc were identified as independent variabls/factors; PS and EE were chosen as dependent variables or responses].

Independent variables	The level used, actual and coded				
	−1.414	−1	0	+1	+1.414
X1 = Span 60 conc (mg) X2 = STC Conc (mg)	64.6447 6.464	75 7.5	100 10	125 12.5	135.355 13.535
Dependent variables					**Goal**
PS (Y1) EE (Y2)					Minimize Maximize

### 2.3 Evaluation and optimization of the prepared FBP-loaded bilosomes

#### 2.3.1 Determination of FBP entrapment efficiency percent:

The determination of the percentage of entrapped flurbiprofen (FBP) was conducted indirectly by assessing the amount of unentrapped FBP in the dispersion medium. A 1 mL aliquot of the dispersion medium underwent centrifugation at 15,000 rpm for 1 h at 4°C using a cooling centrifuge. After centrifugation, the supernatant was carefully collected, while the residue was washed with 10 mL of phosphate-buffered saline (PBS) before undergoing re-centrifugation. The resulting supernatant, containing the unentrapped drug, was then analyzed spectrophotometrically at the wavelength of maximum absorption (λmax) of 376 nm using a Shimadzu UV spectrophotometer. This process was repeated three times, and the mean value along with the standard deviation (SD) was calculated for each determination. The entrapment efficiency (EE%) was determined by subtracting the concentration of free FBP in the supernatant from the total amount of drug initially incorporated. Mathematically, this was expressed using the following equation:

$$\text{EE}\%=\left(\text{Total amount of FBP }‐\text{ Unentrapped FBP}\right)\;/\text{ Total amount of FBP}\times 100$$

#### 2.3.2 Determination of particle size of FBP-loaded bilosomes

The average particle size of the prepared flurbiprofen (FBP)-loaded bilosomes was determined using dynamic light scattering at a temperature of 25°C ± 2°C. A Zetasizer, equipped with a helium–neon laser (Mastersizer 3,000, Malvern Instrument Ltd., Worcestershire, United Kingdom), was utilized for these measurements. Prior to each assessment, the bilosomal dispersions were appropriately diluted with deionized water to ensure that the light scattering amplitude remained within the sensitivity range of the instrument. To ensure accurate and reliable results, the bilosomal dispersions were appropriately diluted with deionized water prior to each measurement. This dilution step was crucial to maintain the light scattering amplitude within the sensitivity range of the instrument. The pH of the deionized water used as the dilution medium was approximately neutral (pH 7.0), which is ideal for maintaining the stability of the bilosomal dispersions during analysis. The analysis time for each measurement was consistently set at 70 s, and three replicates were conducted for each sample to ensure the reproducibility of the results (Aziz et al., 2019; Abbas et al., 2021).

### 2.4 Optimization of FBP-loaded bilosomes

During the optimization process, the Design-Expert^®^ software utilized the desirability function to determine the optimal formulation for bilosomes. The primary objective was to identify flurbiprofen (FBP)-loaded bilosomes with the highest entrapment efficiency and the smallest particle size. Responses closer to a desirability factor of 1 were prioritized. Subsequently, the recommended FBP-loaded bilosomes were formulated, evaluated, and compared with the expected responses to validate the model (El-Ridy et al., 2017; Aziz et al., 2019).

### 2.5 Preparation of superparamagnetic iron oxide nanoparticle

The synthesis of superparamagnetic iron oxide nanoparticles (SPION) followed the coprecipitation technique as described by Abbas et al. Initially, ferrous sulfate tetrahydrate (0.6 g) and ferric chloride hexahydrate (1.17 g) were individually dissolved in 50 mL of deionized water under a nitrogen atmosphere, maintaining a molar ratio of 1:1.75. The solutions underwent vigorous agitation, were mixed at 70°C for 1 h, and then treated with ammonium hydroxide (32%). After an additional hour of stirring, the mixture was allowed to cool to room temperature, resulting in a color change from yellow to black, indicative of magnetite nanoparticle formation. The synthesized nanoparticles were separated from the solution using a magnet, subjected to five washes with hot water, and subsequently dried overnight in a 50°C oven (Natural Convection Oven LDO-080N, Korea).

### 2.6 Preparation of FBP/SPION-loaded bilosomes

The preparation of flurbiprofen (FBP)/superparamagnetic iron oxide nanoparticles (SPION)-loaded bilosomes (abbreviated as FSB) adhered to the previously outlined procedure for FBP-loaded bilosomes, with a minor modification. The dry film of the selected formulation was hydrated using an appropriate volume of PBS/aqueous ferrofluid. Specifically, 700 μL of a mixture containing 10.3 mg/mL of SPION (Abbas et al., 2022) was introduced to achieve a final volume of 10 mL (Chen and Langer, 1997; FFBPis et al., 2011).

### 2.7 Characterization of FSB

#### 2.7.1 Evaluation of entrapment efficiency, particle size, and zeta potential

The entrapment efficiency percentage, particle size, and zeta potential of flurbiprofen (FBP)/superparamagnetic iron oxide nanoparticles (SPION)-loaded bilosomes (FSB) were evaluated using the same methodologies outlined earlier for FBP-loaded bilosomes.

#### 2.7.2 Magnetism evaluation

The magnetization of FSB was assessed at room temperature utilizing a vibrating sample magnetometer (VSM) (Lake Shore Model 7,410, United States).

#### 2.7.3 Morphological screening of FSB

The morphology of the FSB was examined using scanning electron microscopy (SEM) with a Philips XL 30 microscope from FEI Company (Hillsboro, OR, United States). Unprocessed ethylene oxide methyl cellulose (ETO MC) powder and processed NC powder were first adhered to double-sided tape, then placed in a vacuum chamber for 2 min (10−^6^ Pa). Observations were conducted at 15 kV with the samples coated with 30 nm of gold prior to imaging (Albash et al., 2019).

### 2.8 Lyophilization of FSB

Following refrigeration at −18°C for 24 h, the optimized FSB underwent lyophilization. This process entailed freezing at −45°C and subjecting to a pressure of 0.07 mbar for 24 h to yield a powdered form (Ahmed et al., 2020).

### 2.9 Preparation of FSB in situ forming hydrogels

*In situ* forming hydrogels of FSB were formulated utilizing Synperonic™ PE/F 127 as a thermosensitive polymer, in conjunction with hyaluronic acid (HA). The components were dispersed within the chilled bilosomal dispersion and allowed to equilibrate, followed by refrigeration to ensure complete dissolution (Gugleva et al., 2022).

### 2.10 Evaluation of developed in situ forming hydrogel

#### 2.10.1 Assessment of the gelation temperature

The gelation temperature of the *in situ* forming hydrogels was evaluated using the test tube inversion method (El-Nabarawi et al., 2020; Gugleva et al., 2020). Glass vials containing 2 mL of the studied systems were immersed in a thermostatically controlled water bath. The temperature was gradually increased at a rate of 0.5°C/min from 20°C to 40°C. At each temperature, the test tube was rotated to a 90° angle. The gelation temperature was determined as the temperature at which no flow was observed upon inversion of the vial.

#### 2.10.2 Assessment of gelation time

The gelation time of the newly developed FSB *in situ* forming hydrogels was evaluated employing the test tube inversion method. A test tube containing 2 mL of the chosen FSB *in situ* forming hydrogel (Gugleva et al., 2022) was submerged in a thermostatic water bath set at 37°C ± 0.5°C. Gelation time indicates the duration necessary for the formulation to transition from a liquid state to a gel, characterized by the absence of observable flow upon inversion.

#### 2.10.3 Assessment of viscosity and rheological property

The viscosity of the optimized FSB *in situ* forming hydrogel was assessed using a cone and plate viscometer. Apparent viscosity measurements were recorded at 10 rpm under varying temperatures (4°C and 37°C). Rheological properties were investigated by allowing the dispersion to gel at 37°C. Viscosity was subsequently assessed at various angular velocities, and a rheogram illustrating the flow behavior of the formulation was generated (Mohamed S. et al., 2020).

#### 2.10.4 Syringeability study

The syringeability of the developed formulation was evaluated by loading a syringe with 1 mL of the chilled optimized FSB *in situ* forming hydrogel (FSB4c) and applying gentle pressure to the syringe’s plunger (Swain et al., 2019; Polat and Ünal, 2022).

### 2.11 *In Vitro* release study

Each preparation was included in dialysis bags, which were then immersed in phosphate-buffered saline (PBS) at pH 7.4 and stirred in a water bath maintained at 37°C. Samples were collected at regular intervals and the concentration of FBP was assessed spectrophotometrically at a wavelength of 280 nm. This method allows for the precise monitoring of drug release over time, ensuring consistent and reliable results (Abbas et al., 2022; He et al., 2021). Statistical analysis was conducted using SPSS software, while release mechanisms were scrutinized through model equations (Elgendy et al., 2023).

### 2.12 *In Vivo* studies

#### 2.12.1 Animal housing and handling

Wistar albino male rats weighing between 140 and 150 g were obtained from the Animal House of the Cairo Agriculture’s Clinical Laboratory Centre. The rats were housed in controlled environments with a temperature maintained at 24°C ± 2°C and a 12-h light/dark cycle. They had *ad libitum* access to standard food and water. The Institutional Review Board for Animal Research/Studies Animals provided approval for the animal experiment protocols for the subsequent investigations (Approval No. 13-01-2024).

#### 2.12.2 Experimental design of the in vivo study

Arthritis was initiated by administering carrageenan (0.02 mL/joint) via intra-articular injection into the right knees of rats over a span of 10 days (Hansra et al., 2000). The rats were then divided into five groups, with each group consisting of eight male rats.

Group 1: Normal control
Group 2: Positive control (carrageenan group)
Group 3: Rats treated with intra-muscular injection of *in situ* forming hydrogel of the optimized FSB

The treatment was administered for 10 days, concurrent with the carrageenan injection.

#### 2.12.3 Evaluation of joint diameter

While under anesthesia, the thickness of the knee joint was assessed utilizing an electronic digital caliper (Mitutoyo, Japan).

#### 2.12.4 Evaluation of locomotor activity and coordination

Motor activity was assessed by monitoring rat movements in a grid floor activity cage. Rats were acclimatized before being placed in the activity cage (Kauppila et al., 1991; Hashmat et al., 2020), and activity counts were measured in successive sessions. The locomotor activity and coordination were evaluated at the end of the experiment (Edet et al., 2015; Salama et al., 2016). Inflammation using histological analysis of joint tissues. This method involved examining tissue samples for signs of inflammation, such as the presence of inflammatory cells and changes in tissue structure.

### 2.13 Statistical evaluation

The data are expressed as means ± standard deviation of the means (SD). Group comparisons were conducted utilizing one-way analysis of variance (ANOVA), followed by Fisher’s LSD test for multiple comparisons (GraphPad Prism software, version 5 (Inc., United States)). Statistical significance was defined as p < 0.05.

## 3 Results and discussion

### 3.1 Optimization of preparation of FBP-loaded bilosomes

**FIGURE 1:**
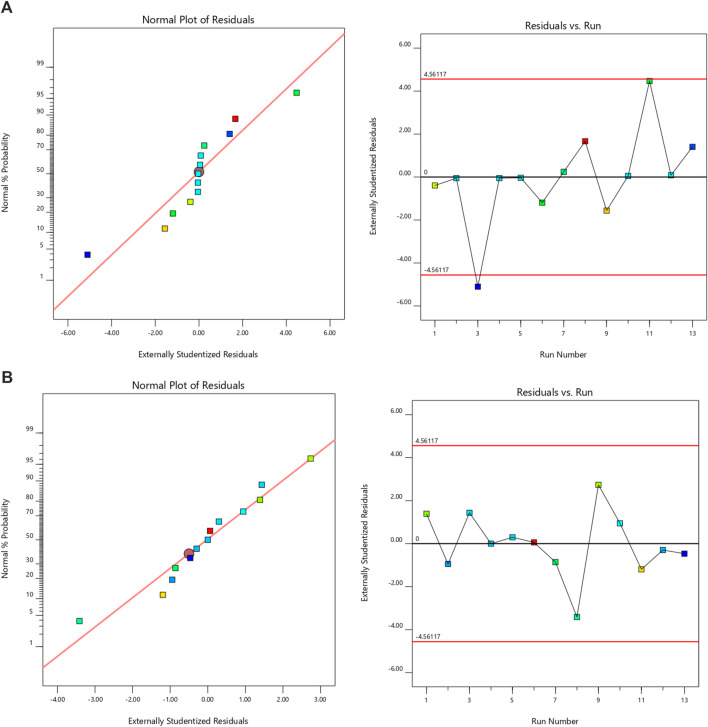
The normal plot for residuals and residuals vs. run for **(A)** PS and **(B)** EE [In Normal plot of resuduals, the colour points represent the formulation trails and they were symmetrically distributed around the red line, indicating that there is no significant skewness in the residuals. In residulas Vs. run, the black horizontal line at zero represents the ideal scenario where residuals are perfectly centered around zero, indicating no systematic error. The red horizontal lines at approximately ± 4.56 represent control limits].

Design Expert 11 employed a 3-factor, 3-level Box-Behnken design to investigate quadratic response surfaces (Version 11.0, Stat-Ease Inc.). The results of 13 runs and the reactions of the created formulations are presented in Table 2. Three-dimensional plots are widely recognized for their ability to evaluate the simultaneous effects of two factors on response and the effects of multiple factors and their interactions. The normal plot of residuals provided further evidence of the accuracy of all selected models. In this case, visual inspection was adequate, rendering the suggested statistical program unnecessary. The proposed model can be statistically accepted, as all studentized residuals for the chosen responses were evenly distributed along a straight line. Figure 1 illustrates the experimental run versus the residuals, aiding in the identification of the underlying variables influencing the responses. A scattered trend within the allowed range was observed, indicating the presence of a time-coupled variable in the background.

**TABLE 2:** Observed responses in for developing and optimizing FBP-loaded bilosomes.

Std	Run	Factor 1	Factor 2	Response 1	Response 2
		A:Span 60	B:STC conc	PS	EE
		mg	mg	nm	%
8	1	100	13.5355	698	76
13	2	100	10	253	45
1	3	75	7.5	25	49
9	4	100	10	253	48
12	5	100	10	254	49
3	6	75	12.5	480	99
2	7	125	7.5	396	61
4	8	125	12.5	988	58
6	9	135.355	10	788	78
11	10	100	10	259	51
5	11	64.6447	10	450	85
10	12	100	10	261	47
7	13	100	6.46447	89	35

#### 3.1.1 Study of effect of independent variables or selected factors on PS (response 1-Y1)

Small particle size distribution is a crucial prerequisite for effective drug delivery. Particle sizes ranged from 25 to 988 nm (Table 2). Typically, the average particle size tends to increase with higher span concentration. Similarly, the size of the bilosomes increased with the rise in STC concentration from 6.46 to 7.5 mg, but decreased when the concentration was further increased from 7.5 to 13.5 mg.

PS=+256.00+169.63A+238.53 B+34.25 AB+173.00 A2+60.25 B2

**FIGURE 2:**
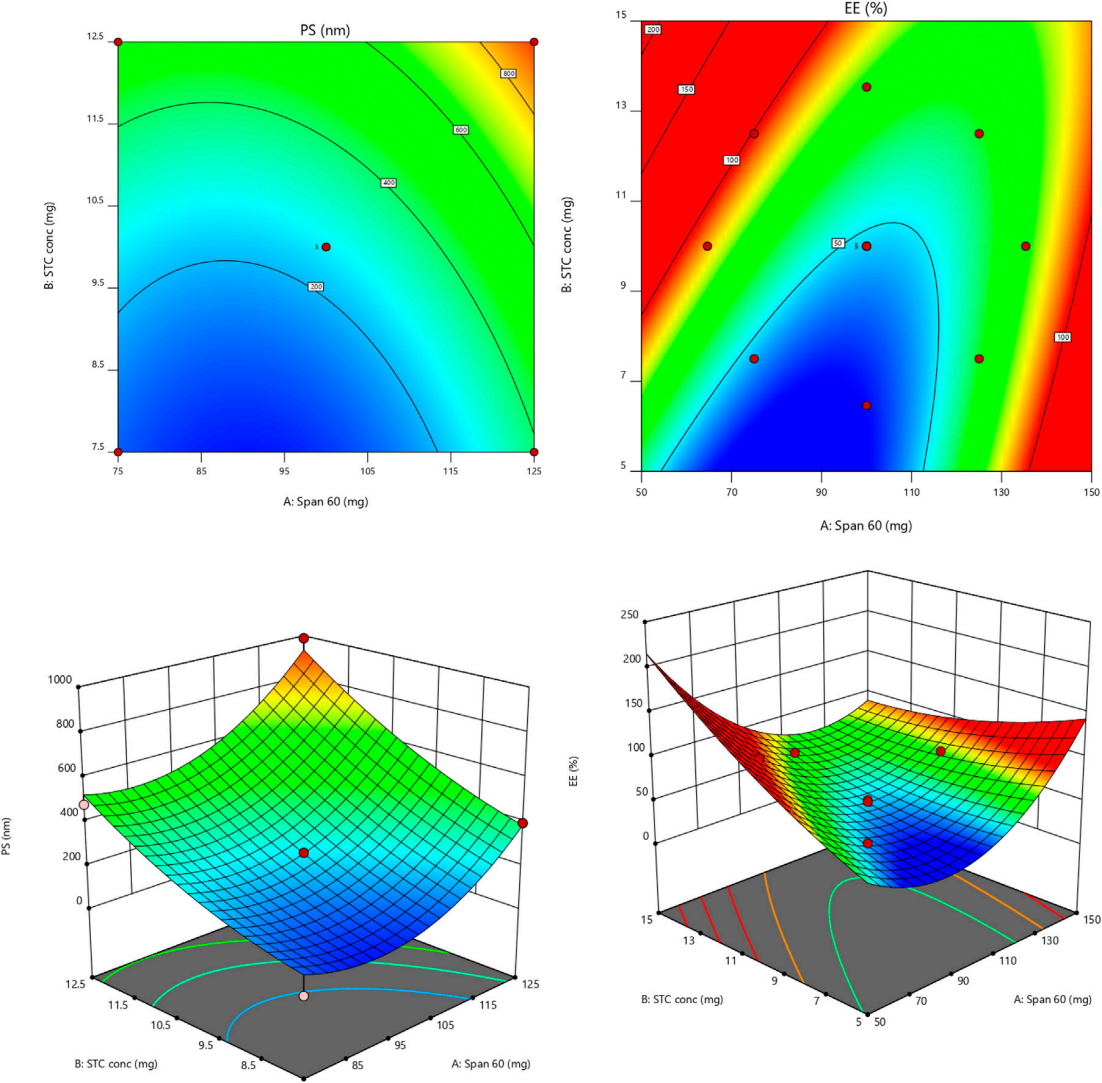
Contour and 3-D response graphs for PS and EE [colour variation shows the changes of responses values from low (blue) to high (red) values].

The model’s F-value of 47.48 is statistically significant, indicating that noise may cause such a high F-value only 0.01% of the time. P-values below 0.0500 signify the necessity of model terms. In this instance, terms A, B, A^2^, and B^2^ are significant [refer to Table 3]. The F-value for lack of fit being 636.29 suggests that lack of fit is not considerable compared to the total error (Bakhaidar et al., 2022; Alhakamy et al., 2022). The small difference between the predicted R2 of 0.7967 and the adjusted R2 of 0.9509, less than 0.2, implies reasonable consistency between the two values. Adeq Precision assesses the signal-to-noise ratio, with a recommended ratio of at least 4. The ratio of 21.2394 indicates an adequate amount of signal (references Alhakamy et al., 2022; Bakhaidar et al., 2022). Residual plots for the responses are depicted in Figure 2, while linear correlation plots (A) compare actual values to predicted values.

**TABLE 3:** ANOVA coefficients table.

Parameters and p-values	Intercept	A	B	AB	A^2^	B^2^
PS	256	**169.626**	**238.532**	34.25	**173**	**60.25**
p-values		**0.0001**	**< 0.0001**	0.3047	**0.0002**	**0.0371**
EE	48	**−4.86244**	**13.1228**	**−13.25**	**16.3125**	**3.3125**
p-values		**0.0059**	**< 0.0001**	**0.0001**	**< 0.0001**	**0.0421**

Bold terms indicate significant terms.

#### 3.1.2 Study of effect of independent variables or selected factors on zeta potential (response 2-Y2)

With an F-value of 66.61, it is presumed that the model is statistically significant, as there is only a 0.01% likelihood of such a large F-value occurring due to noise. P-values below 0.0500 indicate the necessity of model terms. Significant model terms encompass A, B, AB, A^2^, and B^2^. The modest F-value for the lack of fit, just 4.45, indicates that lack of fit does not significantly deviate from the standard error of the estimate.

EE=+48.00 −4.86A+13.12 B −13.25 AB+16.31 A2+3.31 B2

**FIGURE 3:**
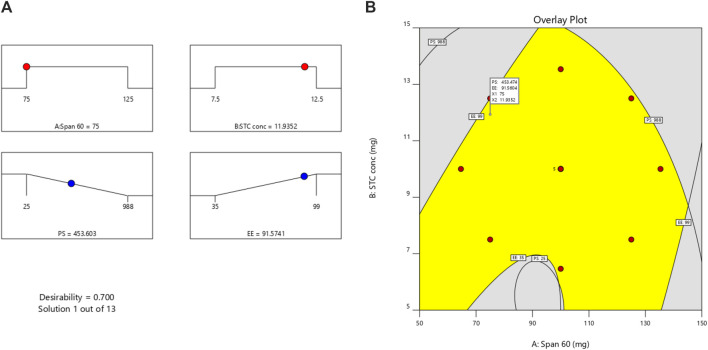
**(A)** Desirability plot and **(B)** overlay plot [overlay of all the factors for getting desired response, Yellow coloured area represents the design space of optimization].

The 3D-Response graph for A indicated that higher span values correlated with increased PS. Both Span and STC exerted significant impacts on PS. While Span concentration had minimal influence on Y2 (EE) response, STC showed significant effects. Utilizing the desirability function [D], optimization of multiple models from the experimental study was feasible. For each response, including PS-minimum and zeta potential-maximum, several constraints were set to generate the overlay graph. All selected variables fell within the design space. At optimal concentrations of independent variables, the combined desirability plots of all responses reached the highest D value of 0.700. Crucial responses were overlaid in the contour plot [Figure 3]. Based on this approach, ideal formulation conditions could be achieved with Span 60 at 75 mg and STC at 11.93. These concentrations are expected to yield a particle size (PS) of 453.60 nm [Supplementary Figure S1] and an entrapment efficiency (EE) of 91.57%. With these anticipated ideal concentrations, a new optimized formulation was developed and evaluated. Experimental results were compared with theoretical values to validate the experimental design, demonstrating under 3% relative inaccuracy.

A zeta potential of 35.9 mV indicates a moderately high negative charge on the nanoparticles, facilitating electrostatic repulsion between particles and preventing aggregation. Enhanced electrostatic repulsion contributes to nanoparticle dispersion in the formulation, bolstering colloidal stability. PDI assesses the average homogeneity of a particle solution, with higher values suggesting a broader size distribution within the particle sample. Additionally, PDI can unveil nanoparticle aggregation and variations in particle surface changes across the sample. The optimized formulation demonstrated an acceptable polydispersity index value of 0.12, indicative of consistent particle size distribution and minimal aggregation (Salama et al., 2022b; Sreeharsha et al., 2022).

#### 3.1.3 Implication of DoE for formulation development

DoE approach played a pivotal role in the optimization of the flurbiprofen-loaded bilosomes (FSB) hydrogel formulation. By employing the desirability function [D], we were able to optimize multiple responses simultaneously, making the process highly efficient and systematic.

#### 3.1.4 Optimization process

Utilizing DoE allowed us to create models for various critical responses, such as particle size (PS) and zeta potential, under a set of predefined constraints. For each response, including PS-minimum and zeta potential-maximum, we generated overlay graphs to visualize and identify the optimal formulation conditions within the design space. At optimal concentrations of the independent variables, the combined desirability plots of all responses reached the highest D value of 0.700. This high desirability score indicated that the selected formulation conditions were the most favorable for achieving the desired outcomes. Specifically, the ideal formulation conditions were determined to be Span 60 at 75 mg and STC at 11.93 mg. These concentrations were expected to yield a particle size of 453.60 nm and an entrapment efficiency of 91.57%, as illustrated in the overlay contour plot (Figure 3).

#### 3.1.5 Experimental validation

To validate the optimized formulation, we developed a new batch based on the identified optimal conditions and compared the experimental results with the theoretical values predicted by the model. The experimental results showed a particle size of 453.60 nm and an entrapment efficiency of 91.57%, closely matching the predicted values. The relative inaccuracy was found to be under 3%, demonstrating the robustness and accuracy of the DoE approach in predicting optimal conditions.

#### 3.1.6 Zeta potential and stability

The zeta potential of the optimized formulation was measured at 35.9 mV, indicating a moderately high negative charge on the nanoparticles. This high zeta potential is crucial for maintaining colloidal stability as it facilitates electrostatic repulsion between particles, preventing aggregation. Enhanced electrostatic repulsion contributes to better nanoparticle dispersion within the formulation, which is essential for maintaining the stability and efficacy of the hydrogel over time.

#### 3.1.7 Benefits of the DoE approach

By leveraging the DoE methodology, we were able to systematically explore the formulation space and identify the optimal conditions for our FSB-loaded hydrogel. This approach not only streamlined the optimization process but also provided a clear understanding of the interactions between different formulation variables. The ability to simultaneously optimize multiple responses ensured that the final formulation met all critical quality attributes, leading to a more effective and stable drug delivery system.

In conclusion, the DoE approach significantly enhanced the formulation development process by providing a robust framework for optimization. The resultant formulation exhibited desirable properties such as controlled particle size, high entrapment efficiency, and excellent colloidal stability, making it a promising candidate for prolonged and controlled drug delivery applications.

### 3.2 SPION and FBP/SPION-loaded bilosomes

The synthesized superparamagnetic iron oxide nanoparticles (SPIONs) displayed a small particle size of 35.0 ± 7.0 nm and a positive surface charge, with a zeta potential of 38.6 ± 0.85 mV. Upon incorporation into optimized flubiprofen (FBP)-loaded bilosomes, a notable increase in particle size to 523 nm was observed, while the zeta potential concurrently decreased to 36.24 mV. This increase in particle size is attributed to the inclusion of iron oxide nanoparticles, whereas the reduction in zeta potential is likely due to the positively charged iron oxide nanoparticles adhering to the bilosomes’ surface, consequently diminishing the vesicles’ negative charge. Importantly, despite these alterations, there was no significant impact on the FBP entrapment efficiency within the bilosomes, indicating the sustained ability of the bilosomes to effectively encapsulate the drug.

### 3.3 Magnetic properties

**FIGURE 4:**
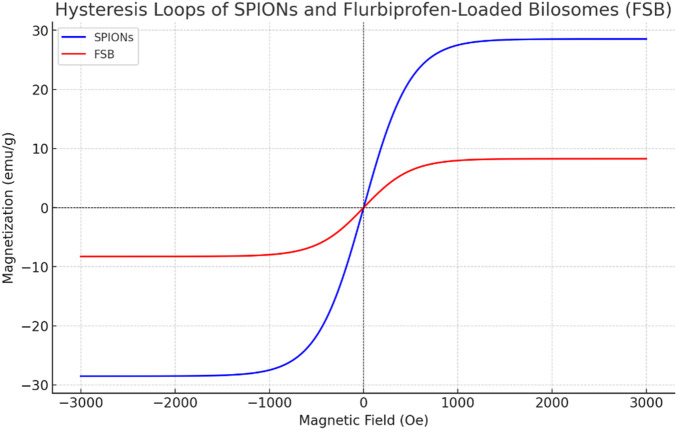
Hysteresis loops of SPIONs.

The saturation magnetization values of superparamagnetic iron oxide nanoparticles (SPIONs) and flubiprofen-loaded bilosomes (FSB) were determined to be 28.528 emu/g and 8.269 emu/g, respectively, based on the hysteresis loops. It was noted that all the magnetic hysteresis curves intersected the origin, indicating zero coercivity and remanent magnetization for both SPIONs and FSB [Figure 4]. This behavior signifies a superparamagnetic nature for both prepared SPIONs and flubiprofen-loaded bilosomes. Despite the saturation magnetization of FSB being lower than that of SPIONs, FSB still exhibited significant magnetic responsiveness to external magnetic fields. This magnetic responsiveness is attributed to the entrapment of SPIONs within the bilosomes. By incorporating SPIONs into the bilosomal structure, the magnetic properties of the nanoparticles are retained even within a different matrix.

Consequently, the entire FSB formulation remains responsive to external magnetic fields. The ability of the entrapped nanoparticles to be influenced by an external magnetic field is a critical feature, enabling targeted delivery of FSB to specific sites within the body by simply applying an external magnetic field. This magnetic targeting capability is essential for drug delivery applications, facilitating precise localization of the therapeutic agent to desired locations. The superparamagnetic nature of both SPIONs and FSB, combined with their entrapment within bilosomes, thus presents opportunities for targeted drug delivery strategies where an external magnetic field can strategically enhance drug accumulation at specific sites, such as inflamed joints in rheumatoid arthritis management (Bakhaidar et al., 2022; Alhakamy et al., 2022).

### 3.4 Morphological screening of FSB

**FIGURE 5:**
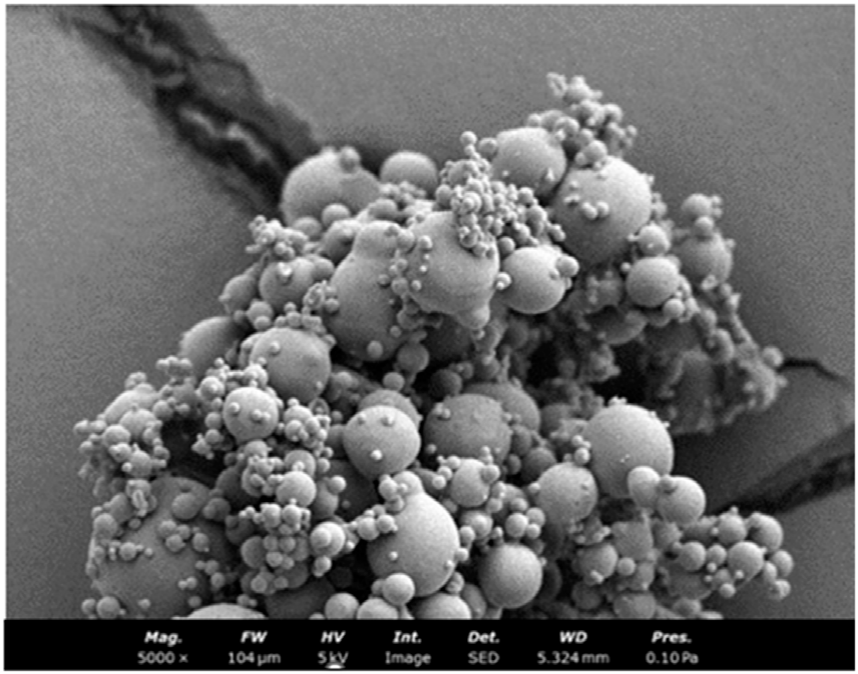
SEM image of nanoparticles.

The SEM images of nanoparticles were spherical globular clusters shape (Figure 5).

### 3.5 Preparation of FSB in situ forming hydrogels

In this study, we opted for the cold method over the hot method for preparing an *in situ* forming hydrogel. This decision stemmed from the improved solubility of the polymer in a cold environment. At lower temperatures, the polymer demonstrated enhanced solubility due to the formation of hydrogen linkages, resulting in the generation of a clear solution. Specifically, we chose Synperonic™ PE/F 127 for its thermo-gelling properties. This polymer remains in a liquid state at low temperatures but undergoes a phase transition to form a gel at higher temperatures.

The optimized formulation of flubiprofen-loaded bilosomes (FSB) in the *in situ* forming hydrogel exhibited a rapid gelation time of approximately 40 ± 1.8 s, with gelation occurring at 37°C ± 0.9°C. These parameters are highly favourable for injection applications. The short gelation time suggests rapid transition from liquid to gel state, which is advantageous for *in-vivo* applications requiring prompt gel formation. Additionally, the gelation temperature of 37°C aligns with the recommended temperature for developing *in situ* forming hydrogel systems intended for injectable use.

The selection of the FSB *in situ* forming hydrogel for further investigation is justified by its adherence to optimal conditions for injectable hydrogel systems. This temperature-responsive hydrogel system holds promise for controlled and sustained drug release, making it suitable for applications demanding precise drug delivery and localized therapeutic effects. Our methodology, involving the cold preparation method and the choice of Synperonic™ PE/F 127, represents a deliberate approach aimed at enhancing polymer solubility and thermo-gelling behavior to successfully develop the *in situ* forming hydrogel.

### 3.6 Assessment of pH, viscosity and rheological properties

The final pH values for each formulation were determined as follows: for the FBP-loaded bilosomes, the pH was approximately 6.0. Upon synthesizing the superparamagnetic iron oxide nanoparticles (SPIONs), the final pH stabilized around 7.0. Integrating SPIONs into the FBP-loaded bilosomes to form FBP/SPION loaded bilosomes resulted in a final pH of about 6.5. Characterization of these FBP/SPION loaded bilosomes (FSB) confirmed their uniformity, stability, and surface charge. Finally, the formulation of FSB into *in situ* forming hydrogels adjusted the pH to approximately 7.4, optimizing the conditions for sustained release and therapeutic efficacy.

**FIGURE 6:**
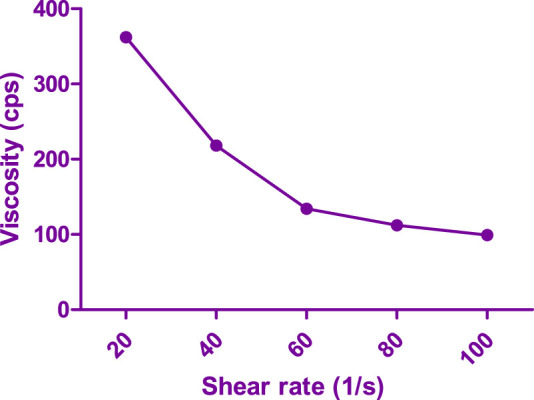
Rheological characterization of FSB loaded hydrogel.

The viscosity of the flubiprofen-loaded bilosomes (FSB) *in situ* forming hydrogel was assessed at 10 rpm, yielding values of 792.6 ± 8.35 cps at 4°C and 332.45 ± 13.25 cps at 37°C. This data illustrates a nearly fourfold increase in viscosity with the transition from 4°C to 37°C, primarily due to the gelation process. Rheological analysis of the optimized hydrogel formulation, particularly the LSB *in situ* forming hydrogel (LSB4c), revealed a shear-thinning pseudoplastic flow, as illustrated in Figure 6. Notably, the pseudoplastic flow observed in the hydrogel offers advantages for injectable preparations. High shear rates during injection decrease the hydrogel’s viscosity, facilitating the injection process. Conversely, at low shear rates, the hydrogel maintains its regular structure, ensuring stability and consistency.

### 3.7 *In-vitro* release study

**FIGURE 7:**
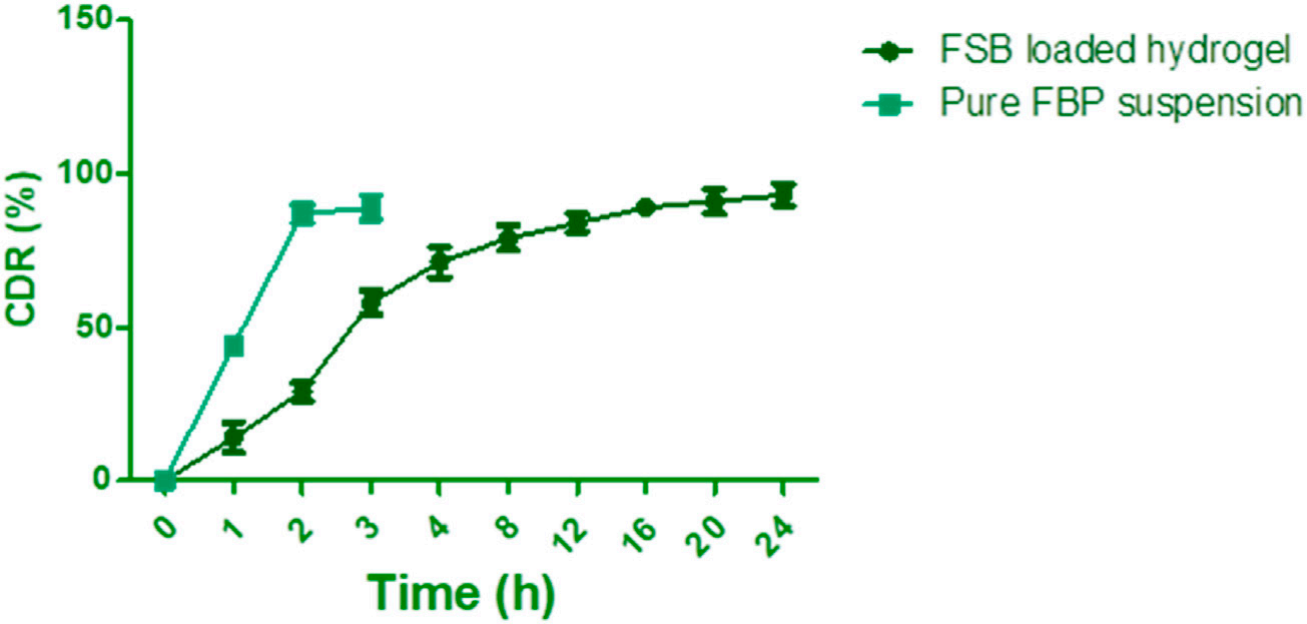
*In vitro* drug release profile of FSB loaded hydrogel. [Data points represent the mean values from three independent samples (n = 3). Error bars indicate the standard deviation (SD) of the mean].

Figure 7 presents a comparative analysis of flurbiprofen (FBP) release from the optimized flurbiprofen-loaded bilosomes (FSB) hydrogel and free FBP suspension. Notably, the free drug suspension demonstrated rapid and complete release, reaching 100% within the initial 3 h. Conversely, the hydrogel formulation exhibited a sustained release profile characterized by two distinct phases. The first phase featured a burst release over the initial 4 h, attributed to the presence of free drug in the dispersion medium and loosely attached drug molecules on the bilosomal surface. Subsequently, the hydrogel transitioned into a second phase characterized by steady and controlled release kinetics. Over a 24-h duration, the optimized hydrogel released 93.24% ± 3.58% of the encapsulated FBP gradually. This biphasic release pattern underscores the role of bilosomes within the hydrogel as drug reservoirs. These bilosomes facilitate sustained and controlled drug release, contributing to the prolonged release profile. The capacity of bilosomes to serve as drug reservoirs is instrumental in achieving sustained release, offering potential advantages for therapeutic applications requiring prolonged and controlled drug delivery to maintain therapeutic concentrations over an extended duration.

To evaluate the statistical significance of the differences observed between the release profiles of the FSB-loaded hydrogel and the free FBP suspension, a two-way ANOVA followed by Tukey’s *post hoc* test was performed. The results indicated highly significant differences at various time points. For instance, at the 1-h mark, the release from the FSB-loaded hydrogel (14.00% ± 5.00%) was significantly lower than that from the pure FBP suspension (44.00% ± 2.00%) (*p <* 0.001). Similar statistical significance was observed at subsequent time points, confirming the distinct release behaviors of the two formulations. The controlled drug release from the bilosomal dispersion within the hydrogel holds paramount importance in optimizing drug delivery systems and enhancing therapeutic efficacy. The statistical analysis supports the observation that the FSB-loaded hydrogel provides a more sustained release compared to the free FBP suspension, making it a promising formulation for therapeutic applications that require prolonged and controlled drug delivery to maintain therapeutic concentrations over an extended duration.

The release mechanism of the drug from the hydrogel formulations was evaluated using the Korsmeyer-Peppas model. The calculated release exponent (n) values for the hydrogel formulations were determined to be ≤0.45. This finding indicates that the drug release mechanism from the hydrogel formulations adheres to Fickian diffusion. Fickian diffusion involves the predominant release of the drug through molecular diffusion, underscoring the significance of drug molecule movement within the hydrogel matrix in driving the overall release mechanism. The observed value of n being less than 0.45 further supports the conclusion of Fickian diffusion, affirming that the drug release from these hydrogel formulations is predominantly governed by diffusion processes.

### 3.8 *In-vivo* studies

In this study, we evaluated the intramuscular injection of both free flurbiprofen (FBP) *in situ* forming hydrogel and the optimized flurbiprofen-loaded bilosome (FSB)-loaded hydrogel in rats with carrageenan-induced joint inflammation. In the group receiving the optimized FBP *in situ* forming hydrogel, an external magnet was applied to the knee to attract nanoparticles to the targeted area, with the aim of enhancing treatment efficacy.

#### 3.8.1 Effect on joint diameter

The study explored the impact of osteoarthritis on the diameter of the right knee joint in rats, revealing a significant increase of 61.85% compared to the normal control group. To address this physiological imbalance, a therapeutic approach involving the intramuscular administration of the optimized FSB (flubriporfen/SPION loaded bilosomes) *in situ* forming hydrogel was implemented. The results demonstrated a remarkable reduction in inflammation induced by carrageenan, with a substantial decrease of 27.83% in the diameter of the right knee joint among treated rats compared to those exposed to carrageenan without any intervention (G2). These findings suggest that incorporating FSB-loaded bilosomes into an *in-situ* hydrogel formulation can have a potential therapeutic effect in alleviating inflammation associated with osteoarthritis in rat knee joints. The results underscore the promising role of FSB in targeted drug delivery, presenting an innovative approach for addressing inflammation related to osteoarthritis.

#### 3.8.2 Effect on locomotor activity

**FIGURE 8:**
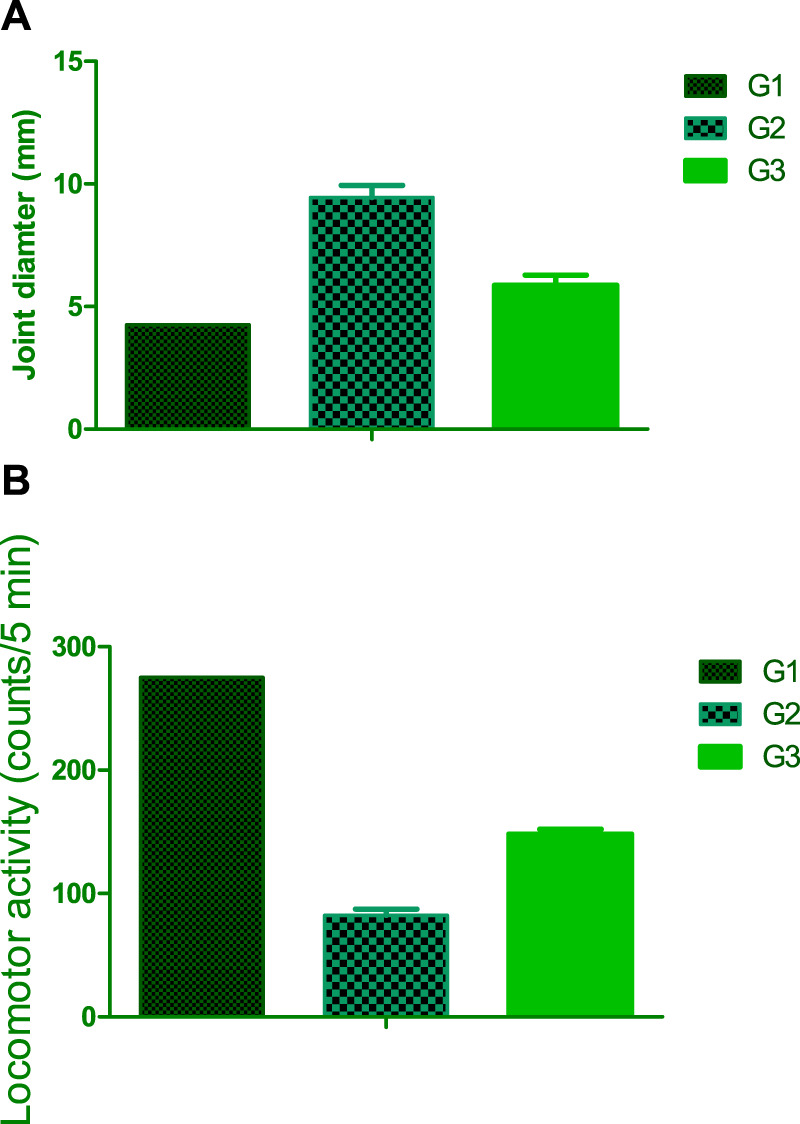
**(A)** Effect on joint diameter **(B)** locomotor activity for G1, G2 and G3.

Osteoarthritis is characterized by a complex dysregulation between chondrocyte synthesis and degeneration processes, resulting in cartilage loss, impaired joint function, and reduced locomotor activity. Experimental induction of arthritis using carrageenan led to a significant decrease in locomotor activity by 72% compared to the control group with normal values. Treatment intervention involved utilizing the optimized FSB (flubriporfen/SPION loaded bilosomes) *in situ* forming hydrogel [Figure 8].

The incorporation of superparamagnetic iron oxide nanoparticles (SPION) within the bilosomal structure holds significant scientific implications in this context. SPION, renowned for their unique magnetic properties, have the potential to facilitate targeted drug delivery to affected joints via an external magnetic field. This targeted approach could enhance the therapeutic effectiveness of loaded drugs like flubriporfen by directing them to the inflamed site in the arthritic joint. Moreover, the bilosomal carrier system, composed of lipid bilayers, provides a biocompatible and stable environment for drug encapsulation, ensuring protection of the therapeutic cargo and enabling sustained release.

The observed increase in locomotor activity by 85% following treatment with the FSB-loaded *in situ* forming hydrogel highlights the potential synergistic effects of SPION and bilosomal delivery in alleviating the impact of arthritis. This improvement suggests a promising strategy for targeted and efficient delivery of therapeutic agents, with implications for ameliorating locomotor dysfunction associated with osteoarthritis. Bhalekar et al. (2017), which investigated Piperine Solid Lipid Nanoparticles (SLN) using a melt emulsification method and demonstrated significant pharmacodynamic responses in topical and oral administration compared to chloroquine suspension. Their findings on TNFα reduction suggest potential DMARD properties of piperine SLN. The integration of SPION within bilosomal structures emerges as a strategic approach to enhance the precision and efficacy of drug delivery for musculoskeletal disorders.

## 4 Conclusion

The systematic optimization of flubiprofen-loaded bilosomes (FSB) using a Box-Behnken design yielded particles with an optimal size of 453.60 nm and an exceptional entrapment efficiency of 91.57%. By incorporating superparamagnetic iron oxide nanoparticles (SPIONs), FSB acquired magnetic responsiveness, enhancing its potential for targeted drug delivery. Comprehensive analyses including morphological examination, magnetic property evaluation, and *in vitro* release studies confirmed the suitability of FSB for controlled and sustained drug release. The formulation of an *in situ* forming hydrogel with FSB showcased promising features, including a rapid gelation time of approximately 40 ± 1.8 s and controlled drug release characteristics. *In vivo* investigations further validated the efficacy of FSB-loaded hydrogel, demonstrating a significant 27.83% reduction in joint inflammation and an impressive 85% enhancement in locomotor activity in osteoarthritic rats. These quantitative findings underscore the potential of FSB as a targeted and effective therapeutic intervention for managing osteoarthritis.

## Data Availability

The original contributions presented in the study are included in the article/Supplementary Material, further inquiries can be directed to the corresponding author.
