# Supplementary material for: Design and evaluation of magnetic-targeted bilosomal gel for rheumatoid arthritis: flurbiprofen delivery using superparamagnetic iron oxide nanoparticles
Source: Front Pharmacol. 2024 Aug 23;15:1433734. doi: 10.3389/fphar.2024.1433734 (PMC11377347; doi:10.3389/fphar.2024.1433734)
Supplement: Supplementary file 1 [file DataSheet1.PDF]

Particle size distribution (intensity)

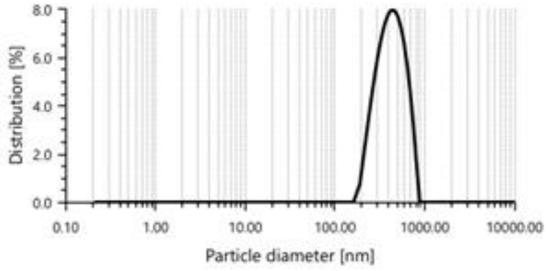

Results

**Hydrodynamic diameter**	453.7 nm	**Mean intensity**	346.4 kcounts/s
**Polydispersity index**	25.5 %	**Absolute intensity**	2565.0 kcounts/s
**Diffusion coefficient**	1.1 µm²/s	**Intercept g1²**	0.9059
**Transmittance**	85.5 %	**Baseline**	1.011

Particle size distribution peaks (intensity)

Peak name	Size [nm]	Area [%]	Standard deviation [nm]
Peak 1	438.4	100.00	150.06

Supplementary Figure 1: Particle size distribution of FBP-loaded bilosomes
